# Improving Composite Tensile Properties during Resin Infusion Based on a Computer Vision Flow-Control Approach

**DOI:** 10.3390/ma11122469

**Published:** 2018-12-05

**Authors:** Juan-Antonio Almazán-Lázaro, Elías López-Alba, Francisco-Alberto Díaz-Garrido

**Affiliations:** Departamento de Ingeniería Mecánica y Minera, Campus Las Lagunillas, Universidad de Jaén, 23071 Jaén, Spain; elalba@ujaen.es (E.L.-A.); fdiaz@ujaen.es (F.-A.D.-G.)

**Keywords:** composite, tensile, optimization, automotive, lightweight design

## Abstract

Liquid composite manufacturing techniques, mainly applied in the transport industry, have been studied and optimized for decades while defect analysis and its minimization have been a goal to increase reliability and mechanical performance. Researchers have found that many process parameters have a strong influence on the mechanical behavior of composite structures where the flow front velocity, closely related to voids, plays a considerable role. In this work, the optimal flow front velocity was evaluated and controlled using a computer vision system for different laminates improving the mechanical tensile properties and void content. Enhanced mechanical tensile properties were found using a feedback flow-controller vision system which was able to keep the optimal flow front velocity constant to reduce the air traps among tows and fibers. Tensile strength was enhanced up to 18% for fiber orientation at 0° and 3.3% at 90°, whereas tensile modulus was increased up to 18.4% for fibers at 0° and 8.7% at 90°. A novel methodology is presented through this work, aiming to improve the robustness of resin film infusion (RFI) processes while ensuring the quality of the composite material.

## 1. Introduction

Composite materials are widely used in aeronautics, aerospace, and the maritime transport industry, and more recently in energy-related industries. In the particular case of the automotive industry, commonly based on aluminum and light alloys, it is now evolving toward electric propulsion [1,2]. In this context, the high specific mechanical properties of composites are playing a considerable role in reducing vehicle weight and minimizing emissions. These goals should be followed by low manufacturing cycle times, cost-effective techniques, repeatability, and high-quality final parts to make production highly competitive [3,4]. Liquid composite molding (LCM) processes, and more specifically vacuum-assisted techniques such as resin film infusion (RFI), are well established to manufacture complex structures in the airplane and naval transportation industries due to their high drapability, low cost, and suitability for producing large structures [5,6] and carrying out repairs [7,8] or for joining purposes [9]. Current knowledge about the manufacturing process, its control, and its automation makes it possible to optimize the composite structures manufactured by these techniques.

The RFI process normally employs one-sided molds in which a bag acts like a mold counterpart, which is highly cost-effective when manufacturing large parts. A large fiber volume fraction is usually reached by this process. In contrast, parts produced by RFI have only one high-quality face and relatively poor quality on the opposite one. As is known, worker skills and know-how influence to a large extent these kinds of semi-manual processes [10].

Composite materials show unavoidably internal and external defects and imperfections, which are mostly generated during manufacturing processes [11]. Most voids or air traps appear during the manufacturing processes, and for critical uses, low percentages are allowed depending on the application [12,13]. Many researchers have shown that these voids or traps have a strong negative influence on the mechanical properties [14,15], acting as stress concentrators and crack initiator points [16]. They appear in different ways and their quantity, shape, and size distribution depend on several factors such as vacuum pressure, inlet pressure, flow front velocity, and mold temperature, among others [17]. Liu and Chen [18] have summarized the causes of void generation during LCM and autoclave processes, including process parameters such as mold temperature, flow front velocity, or packing pressure, resin system features such as surface tension or viscosity, and fabric features such as stacking sequence or aerial density. These parameters have a strong influence on void generation mechanisms such as mechanical entrapment at the flow front, gas entrapments generated during curing, volume changes during curing and shrinkage, or bubble nucleation from dissolved air [19,20]. The state-of-the-art in void content and its actual importance in industrial applications were summarized by [21], where the researchers remarked that void content reduction was a hard challenge. Current applied solutions to reduce void content range from finding optimal gate and vent positions and using wetting agents to degassing the resin prior to injection, increasing the packing pressure during the curing phase, or purging the resin through the vents after injection despite the remarkable effect of uncontrolled flow front velocity, which is usually not expected.

Voids are strongly dependent on how dry fibers get wet, especially in dual-scale fabrics, in which individual fibers are grouped and arranged in tows. Fibers are impregnated by capillary effects while tow spaces are filled by viscosity effects [22], and both define the microflow and macroflow, respectively. In this type of fabric, microscopic pores are generated among the filaments in the fiber tows while macroscopic pores appear among the tows. The unbalance between the macroflow and microflow and its relation to the capillary number have been reported in resin transfer molding (RTM) as one of the most important causes [23,24,25]. In dual-scale fabrics, the modified capillary number is the main parameter to define air trap probability through the relation between capillary and viscosity forces [26]. It has been reported that very low or very high capillary number values mean a viscosity-controlled or a capillary-controlled flow, whereas an optimum-intermediate value tends to equalize the impregnation process in which the flow front velocity is the main controllable and optimizable parameter.

The novel methodology proposed in this work aims to control the pressure loss depending on the real-time flow front velocity while the dry fibers are being impregnated. Thus, a constant and homogeneous velocity through the whole laminate is achieved. The vision system being used allowed the monitoring of the flow front during the RFI manufacturing process using specifically developed algorithms. Once the optimum flow front velocity was known for the specific laminate, a valve-controlled and computer vision system was able to maintain this optimum value throughout the whole process. Therefore, the mechanical properties of the laminate can be improved as a result of the manufacturing process.

The main goal of the novel proposed methodology is to introduce a controllable pressure loss depending on the instantaneous flow front velocity, in order to guarantee a constant and homogeneous flow front velocity while the dry fibers in the whole composite domain are impregnated.

## 2. Fundamentals and Theory

The flow front velocity profile in RFI processes depends on the flow position due to the pressure loss, increasing at the same time as the flow front position is going ahead and covering the dry fibers. Since the total pressure difference applied between the inlet and the outlet is always the same during the whole impregnation process in RFI, it is possible to introduce a controllable pressure loss aiming to keep a constant flow front velocity while dry fibers are impregnated. The Darcy model [27] for porous media (Equation (1)) can be applied to RFI considering a negligible thickness variation [28]:

(1) $$\begin{array}{c} \;u=\frac{\Delta P_{pump}}{\Delta x}\cdot \frac{K}{\mu }\;\to \;u\propto \frac{1}{\Delta x}\\ (\mathrm{for}\;\mathrm{constant}\;K,\;µ\;\mathrm{and}\;\Delta P_{\mathrm{pump}}), \end{array}$$

where *u* is the flow front velocity, *K* is the fabric permeability, and *µ* is the resin dynamic viscosity. Working at usual conditions, the total vacuum pressure applied, named Δ*P_pump_*, is usually near 1 atm depending on the process conditions and the vacuum system capability. From the Darcy model, the flow front velocity and instantaneous position can be obtained as time dependent (Equation (2)):

(2) $$u^{2}=\frac{\Delta P_{pump}\cdot K}{\mu \Delta t}\;\;\mathrm{or}\;\mathrm{in}\;\mathrm{terms}\;\mathrm{of}\;\mathrm{the}\;\mathrm{flow}\;\mathrm{position}:\;x^{2}=\frac{\Delta P_{pump}\cdot K}{\mu }\cdot t.$$

This means that the square of the flow position increment is directly proportional to the time increment when it is plotted in a *x*^2^*-t* graph. Additionally, if viscosity, total pressure, and length are all known, the permeability can be obtained from the plot slope by performing a linear least squares fitting and both process time and velocity can be predicted. As described above, when an additional variable pressure loss is introduced into the system through a valve, Δ*P_valve_*, the flow front velocity can be modified as indicated in (Equation (3)):

(3) $$u=\frac{\Delta P_{pump}-\Delta P_{valve}}{\Delta x}\cdot \frac{K}{\mu }$$

The flow front velocity is at a maximum when the valve is totally opened (Δ*P_valve_ =* 0) and zero when it is totally closed (Δ*P_valve_ =* Δ*P_pump_*). When no valve is present, the flow front velocity is at the maximum possible velocity (*u_max_*(*x*)), whereas when a valve control is present, *v_with C_* (*x*) is lower than *u_max_*(*x*), since Δ*P* is always a positive value. Because the impregnation velocity is continuously decreasing, the flow front velocity at the end of the non-controlled process (*u_w/o C at end_*) should be higher than the optimum assessed velocity value (*u_with C_*). Otherwise, the optimum flow front velocity cannot be set for the whole laminate impregnation, even if Δ*P_valve_* is null (Equation (4)):

(4) $$\begin{array}{c} \begin{array}{c} u_{w/o\;C}=\frac{\Delta P_{pump}}{\Delta x}\cdot \frac{K}{\mu }\\ u_{with\;cC}=\frac{\Delta P_{pump}-\Delta P_{valve}}{\Delta x}\cdot \frac{K}{\mu }\; \end{array}\}\;u_{with\;C}-u_{w/o\;C}=\frac{\Delta P_{valve}}{\Delta x}\cdot \frac{K}{\mu }\\ \Delta P_{valve}>0\;\;\;\to \;\;\;u_{w/o\;C}\left(t\right)>u_{w\;C}\left(t\right)>u_{w/o\;C\;at\;end} \end{array}$$

In this context, the flow front velocity can be controlled through a controllable induced pressure loss, aiming to reach a constant flow front velocity which enhances the final material quality, as is shown in this work.

## 3. Materials and Methods

To find a relationship between the flow front velocity, mechanical properties and voids, tests at different fiber orientations were performed. Once the optimum velocity was known, three validation tests were launched in order to correlate and verify the results. This planning was performed for three different laminates: 0°, 45°, and 90° in which glass fibers were impregnated with unsaturated polyester resin in order to find the optimal values of each orientation and the differences between them.

**Figure 1:**
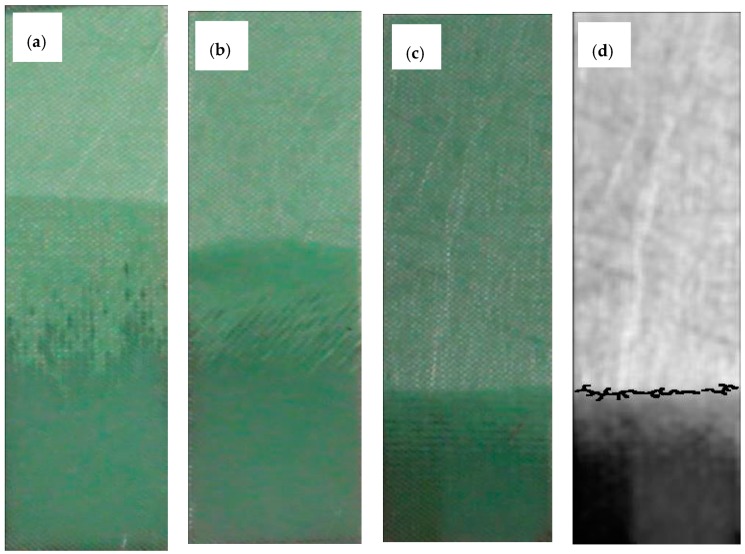
Examples of the specimen impregnation process at different fiber orientations. (**a**) Fibers at 0°, (**b**) fibers at 45°, (**c**) fibers at 90°, and (**d**) flow front recognition for the given specimen at 90° in (**c**).

Preliminary void and mechanical property evaluations were performed at different flow front velocities in order to assess the optimum value. The vision system allowed the monitoring of the flow front in real time during the RFI manufacturing process at different fiber orientations using a computer and specifically developed algorithms (Figure 1).

**Figure 2:**
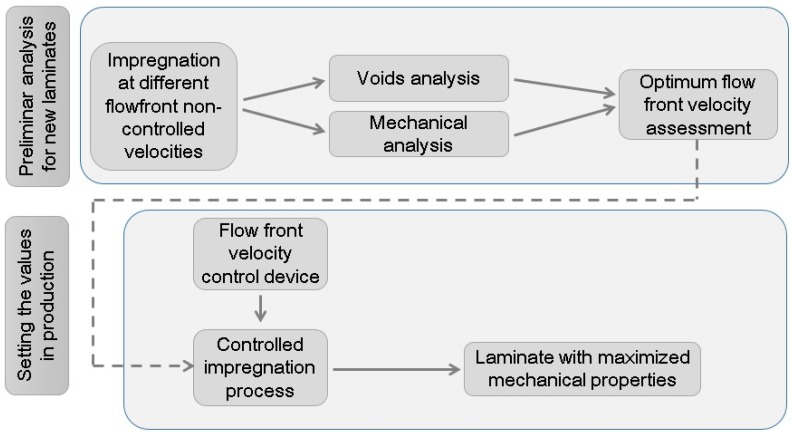
Methodology scheme summary from which an optimum flow front velocity is assessed, aiming to be applied in the production of a specific laminate.

Once the optimum flow front velocity was known for the specific laminate, a valve controlled by a Proportional-Integral-Derivative (PID) system and artificial vision was able to keep this optimum value throughout the whole process. The process is summarized in Figure 2.

### 3.1. Manufacturing Process Description

**Figure 3:**
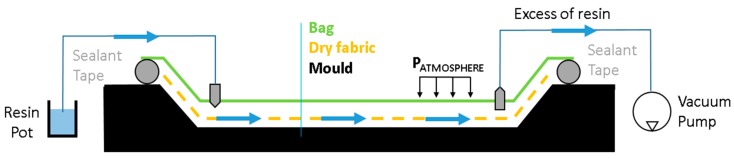
Schematic illustration showing the resin film infusion (RFI) process, where the mold (in black) is covered by the fabric and both are hermetically closed by a vacuum bag surrounded by sealing tape. The resin input is placed on one side (on the left) and the vacuum port is on the opposite side (on the right).

In the RFI method, once the wet laminate is placed over a one-sided steel sheet mold, a sealant tape is also used to seal the bag around the tool periphery, making the assembly impermeable. A vacuum port is placed on one side of the bag and the vacuum is applied while the resin pot is connected to the bag on the other side, as is shown in Figure 3. Once the vacuum is applied at the vacuum port, the atmosphere pressure acts all over the laminate surface and the free surface of resin in the pot, achieving a prior resin absorption effect and a later packing effect over the laminate placed into the bag. Thus, the resin wets the fabric until the flow reaches the vacuum port and the inlet valve is turned off. A peel ply is also included at the top of the laminate to make the flow media release easier. An additional high porosity layer (Dianet, with 135 g/m^2^ and 1.19 mm of thickness) was also added to the top of the laminate to increase the fabric permeability [29], as is usually done in industrial RFI processes. The vacuum bag being used was a 7400 nylon-based with 75 µm (model BF-32 Wrightlon^®^) (Airtech). Diadrain 50 × 4 mm plain channels with Ø9–Ø12 mm hoses and T-connections made of polyethylene were also employed for the resin/vacuum inlets. The pipe length was minimized to reduce the pressure losses along them. A two-stage vacuum pump (Edwards^®^ 80) (Airtech Inc., Huntington Beach, CA, USA) was also employed for manufacturing. The adopted mold was a 900 × 700 mm aluminum sheet, containing all the different laminates to be impregnated in completely independent 300 × 300 mm cavities with different sealed bags and independent valves to control the flow for each of the laminates.

A unidirectional large fiber reinforcement E-type of 600 g/m^2^ was chosen for all tests. It was configured in individual fibers gathered in tows and joined by non-structural polyester yarn in a weaving form with 8.25 g/m^2^. As is usually done in glass fiber, the ply at the top and at the bottom of the stacking was a continuous strand mat E-type of 300 g/m^2^ to homogenize the surface texture. The resin system was an unsaturated polyester resin based on Palatal^®^ P 4 TV-28 (Aliancys, Shaffhausen, Switzerland) and a medium reactivity catalyzer Curox^®^ M312 (United Initiators, Pullach, Germany) (2% in weight) with a dynamic viscosity of 335 mPa·s at room temperature.

The resin was degassed in all cases to reduce the void formation risks even though some authors such as [22] have reported that there is no influence from the resin system degassing on macrovoid formation. The significant influence of vacuum and curing pressure on void content is well known [30,31,32]. In order to avoid these effects, all manufactured laminates were cured at the maximum vacuum level, −95.0 ± 0.1 kPa manometric pressure. The vacuum pressure during the infusion process was also fixed at this value. The valves in the setup were installed to control the opening at the beginning of process. Total agreement is lacking about the influence of curing temperature on void formation, although some authors conclude that it could affect the mechanical properties of the laminate [33,34]. The curing temperature was also kept at room temperature and it was registered continuously.

A pre-curing phase was done inside the mold during 10 h and a post-curing step was also done out of the mold during 72 h at 25 °C. The room temperature was also monitored during the impregnation process: 25 ± 0.1 °C and 60 ± 0.1% of relative humidity were reported as mean values.

### 3.2. Evaluation of the Optimal Flow Front Velocity

**Figure 4:**
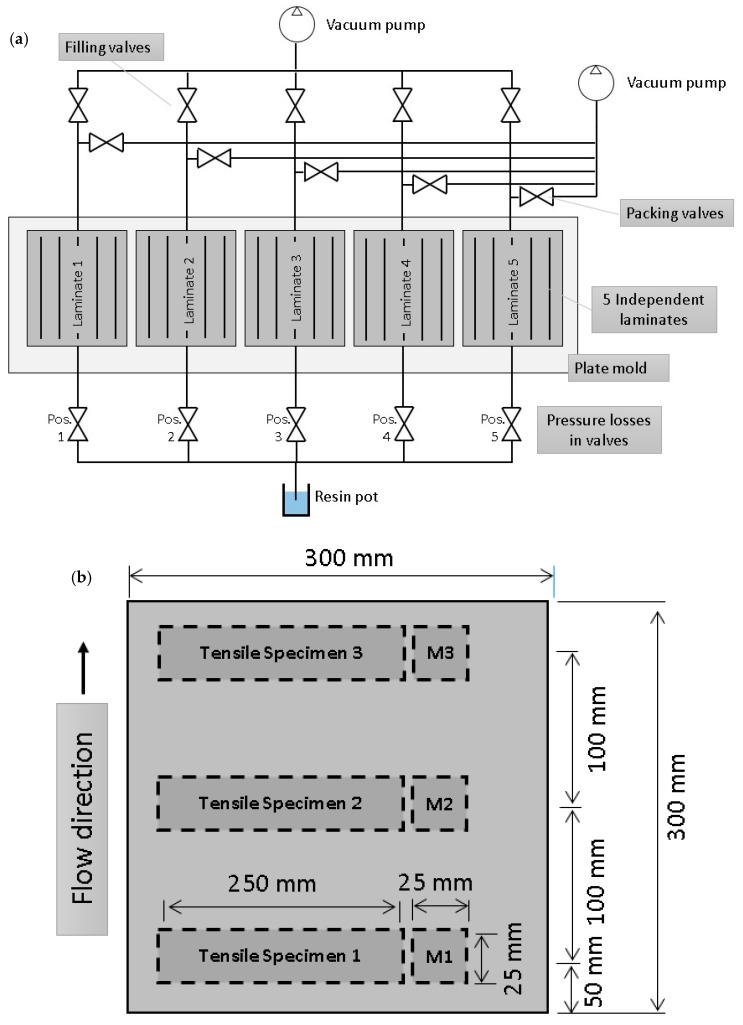
Schematic illustration showing the setup to manufacture the specimens. (**a**) Five laminates were manufactured for each orientation (0°, 45°, and 90°); (**b**) Each laminate was divided into two separate specimens for tensile test and microscopy analysis (M1, M2, and M3), cut at different distances from the resin inlet port (50 mm, 150 mm, and 250 mm).

**Figure 5:**
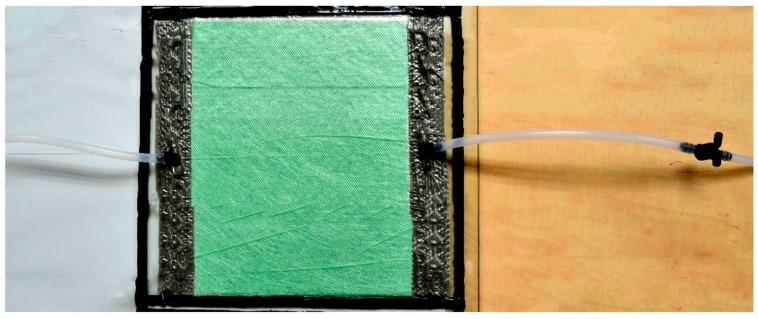
Setup example of the manufacturing process. Valves in the resin inlet and vacuum point can modify the flow front velocity.

Five tests for each orientation were performed to find the optimal flow front velocity, and consequently the value at which optimal mechanical properties were achieved. To avoid uncontrollable influences over the test specimens, all were prepared simultaneously using the same material. The adopted setup made it possible for each laminate to be filled completely (Figure 4 and Figure 5). To obtain homogeneous mechanical properties along the test specimens, the infusion direction was always perpendicular to the unidirectional tensile test. Otherwise, the flow front velocity would be different even for the same test specimen, invalidating any experimental result.

The valves were used to regulate the flow rate in each case introducing a controlled pressure loss into the flow path. One valve was set to 100% opened to assess the maximum flow front velocity which allowed the authors to estimate the lamina permeability for each fiber orientation. The other four valve positions were randomly selected. In this case, the flow rate was a consequence of these values which was subsequently measured by artificial vision. The pressure losses through the whole RFI system was assessed, and, taking into account the high pipe diameters and low flow rates, the pressure losses through the pipes were considered negligible compared to the pressure losses through the saturated laminate ($q\ll \;\;\to \;\;\Delta P_{installation})$. The valves were initially opened very little before starting the tests to fill the pipes. A digital vacuum sensor SMC^®^ - Z/ISE40A-PNP (SMC, Tokyo, Japan) was placed in the vacuum line to monitor the filling and packing phases. It made it possible to detect any irregular behavior during filling.

### 3.3. Flow Front Velocity Control System

During the manufacturing process, image acquisition and digital image processing techniques were employed as non-intrusive techniques to measure and control the flow front velocity. For this purpose, an electromechanical system based on a valve was developed. The flow rate was modified according to the online measured velocity. The system was able to automatically set the flow rate instantaneously to the set point value depending on the flow front position-time read by the mage acquisition system. The optimal flow front velocity was given as an input, depending on the laminate characteristics, which were previously assessed through mechanical and microscopy evaluation, as shown in this paper. Once the resin was going through the dry laminate, a difference in color, contrasts, and brightness of the laminate was detected by the camera. Images were continuously analyzed by a computer to assess the flow front position in real time. It made it possible to determine the instantaneous flow front velocity assessment and valve position change by comparing the measured value with the set point (optimum value of flow front velocity). These variations were managed by an internal PID (Proportional-Integral-Derivative) control.

**Figure 6:**
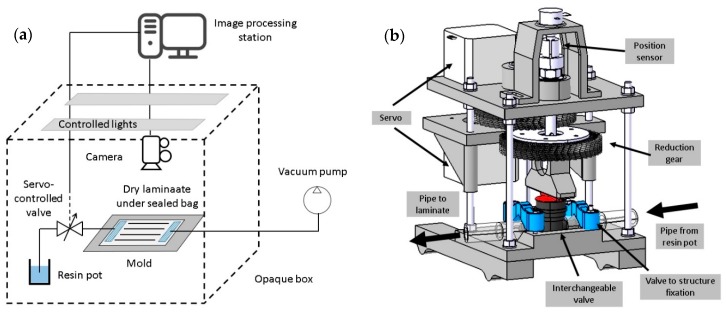
Schematic illustration showing the adopted device for the flow front velocity control system. (**a**) Setup where the laminate and the camera are isolated in an opaque box with the artificial vision system continuously detecting and adjusting the flow front velocity); (**b**) Illustration of the servo-controlled valve employed to adjust the flow front velocity in real time.

In the setup, as shown in Figure 6, the mold and the laminate were inside an opaque box which prevented external lighting influences. Two controlled 10W LED tubes Master LEDTube^®^ 600 mm (Philips, Amsterdam, Netherlands) illuminated the area of interest inside the box improving the performance of the flow front detection algorithm. 

### 3.4. Evaluation of the Mechanical Properties 

Tensile test specimens were manufactured according to ASTM D3039 [35], with a length of 250 mm and a width of 25 mm, overlapped with tabs of 50 × 25 mm made of glass fiber and polyester and bonded with cyanoacrylate-based adhesive. The fiber orientation was defined by modifying the cut direction in the individual laminas before placing them on the mold. Tests were conducted using a universal servo-hydraulic testing machine (model MTS^®^ 370.02, MTS Systems Corporation, Eden Prairie, MN, USA) with a maximum load capacity of 100 kN. A displacement rate of 2 mm/min was set. A Digital Image Correlation (DIC) (Vic-2D by Correlated Solutions, Inc., Irmo, SC, USA) was employed to measure displacements during the tests [36,37,38] using a 1.0 Mpixels monochromatic CCD camera model Guppy Pro^®^ (Allied Vision Technologies, Stadtroda, Germany) with a 23 mm focal length lens Schneider, Bad Kreuznach, Germany). A datalogger unit DAQ T8D-16 (National Instruments, Austin, TX, USA) was also employed to measure the load cell signal. Since the specimen thickness was not uniform due to the manufacturing process, three local measurements were taken in each specimen to evaluate the mechanical properties according to the minimum cross area.

### 3.5. Voids Evaluation

**Figure 7:**
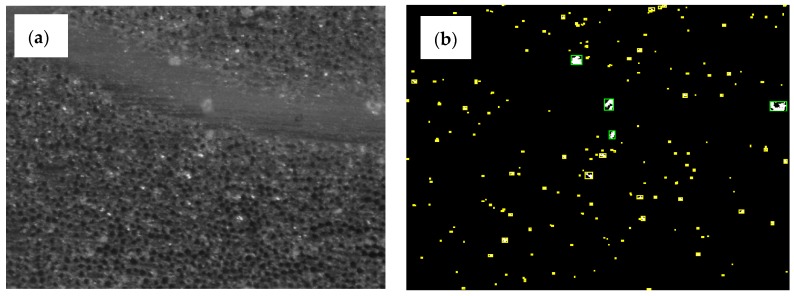
(**a**) Acquired image from microscope analysis and (**b**) processed image including microvoids and macrovoids.

Optical microscopy has been evaluated to perform voids analysis. Some authors [32,39,40] have used this simple technique, finding accurate results compared to other different methods in which digital image analysis helps to quantify voids. Following the procedure described by [39], the samples were cut using a low velocity saw to avoid excessive damage of the glass fibers [41]. Specimens for the microscope were cut from the same laminate as tensile specimens, as shown in Figure 4 and named as M1, M2, and M3. The specimens’ size was set at 25 × 25 mm, as shown in Figure 4. The samples were prepared sliding grain papers over a flat surface to achieve a plain cross-section of the interest area. The surfaces were polished using sandpaper in gradual transition from 100 grains/cm^2^ to the finest of 2500 grains/cm^2^. Before performing microscopy, a polish treatment was applied to obtain a mirror-like finish. Due to the fact that all laminates had unidirectional laminas, microscopy specimens were prepared with respect to two perpendicular directions, one of them parallel to the flow front direction. Additionally, snapshots were taken of the sections in two different positions to cover rich-fiber areas and rich-matrix areas which made the evaluation of macrovoids and microvoids easier afterward. Artificial vision was employed to evaluate the void size distribution and quantity (Figure 7). Some visual adjustments were applied to have the best contrast between voids and material before black and white conversion.

For the microscopy analysis, a Leica M205C (Leica Camera AG, Wetzlar Germany) with a digital camera Leica DFC495 and a lens FusionOptics (Leica Microsystems AG, Wetzlar, Germany) were employed. Each individual image covered an area of 1480 × 1530 µm^2^ with a spatial resolution of 1.00 µm/px. An overlap of 1/3 of the total image width was adopted. The complete assembled image covered 1.5 mm × 2.6 mm approximately. To assess the void content from the images, a specifically developed Matlab algorithm was adopted, following the procedures reported by other authors [40], in which images were segmented using an intensity threshold manually adjusted due to the light condition at each specimen. Then, an object detection was performed and voids smaller than 2 pixels were skipped before quantifying the area covered by voids. A visual inspection of each image helped to verify the void content, located at individual fibers or tows.

## 4. Results and Discussion

The manufacturing RFI process was first evaluated to determine the flow front velocity at the positions in which specimens were cut. Results such as tensile properties and void content were related with the assessed flow front velocity. Five different valve positions were tested for 0°, 45°, and 90° fiber orientations. Three specimens were cut from each laminate; thus, a total of 45 specimens were analyzed. 

### 4.1. Impregnation Analysis

**Figure 8:**
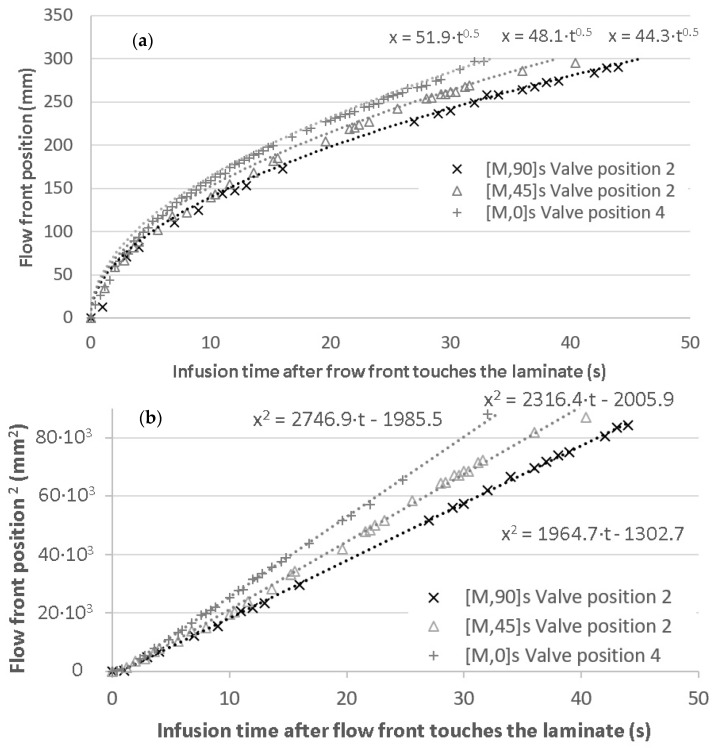
(**a**) Flow front position time-dependent and (**b**) square of flow front velocity time-dependent when the valve is totally opened and a vacuum of −95.0 kPa is applied.

Initially, the filling pattern was evaluated through the flow front position depending on the process time. Figure 8 shows the front position versus the square of flow front position as a time-dependent function when no pressure losses were applied. It is possible to assess the fiber permeability from Equation (2), which is shown in Table 1. As expected, the fabric permeability is clearly dependent on the fiber orientation and, consequently, the filling time is increasing with the fiber angle. The velocity at the end of the process is reduced by 19% from 0° to 45° and around 30% from 0° to 90°. The flow front finds low pressure paths in the spaces between tows, and even between fibers when the fiber orientation coincides with the local flow direction. On the other hand, the flow front finds it difficult to cross through perpendicular fibers, and pressure losses are higher and cutting down the permeability. These values are interesting since they mean a limit in the velocity regulation range because the maximum velocity is imposed by the laminate characteristics.

The minimum flow front velocity at the end of the filling process defines the range in which the system can regulate the flow front velocity for each particular configuration. By using the Darcy model, it was observed that the maximum velocity occurring at the beginning of the filling process did not depend on the laminate features since the pressure losses of dry fiber could be neglected. Due to permeability, the maximum velocity was dependent on the flow front direction relative to the fiber direction. For this purpose, three different fiber directions were evaluated. The vision system allowed the measurement of the permeability using the Darcy model (Equation (2)) for porous media.

**Figure 9:**
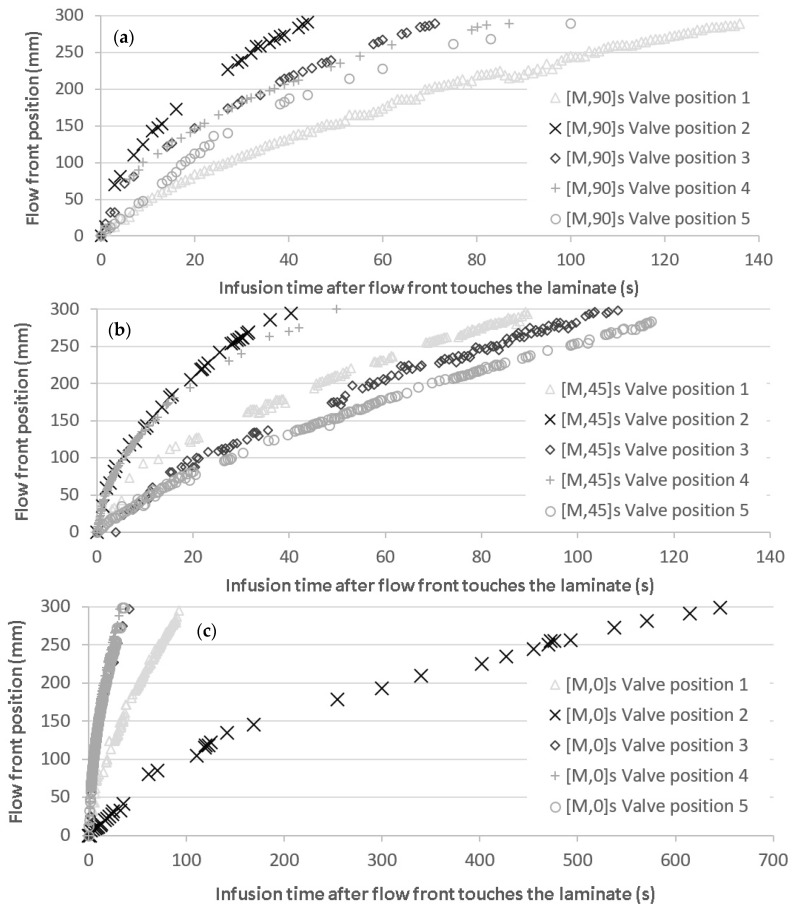
Experimental flow front position for infused laminates at (**a**) 0°; (**b**) 45°, and (**c**) 90° with respect to the fiber direction at different induced velocities.

Modifying the valve positions, different velocity patterns were found for each fiber orientation, as is shown in the three graphs in Figure 9, where the five different valve positions were randomly selected for each orientation. Velocity values at positions described in Figure 4b were collected for comparison of void content and tensile properties.

### 4.2. Relationship between Mechanical Properties and Flow Front Velocity

**Figure 10:**
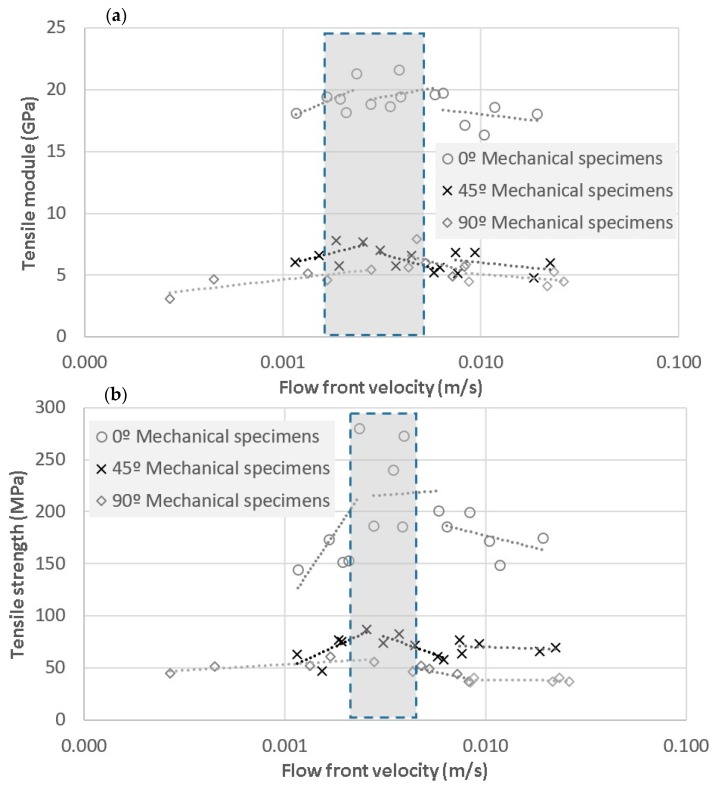
(**a**) Tensile longitudinal strength and (**b**) tensile longitudinal modulus of mechanical specimens depending on the flow front velocity for laminates impregnated at 0°, 45°, and 90°. The shaded area represents the range of flow front velocities in which the highest properties were founded.

Tensile specimens were tested following ASTM D3039. The tensile modulus and tensile strength are plotted against the flow front velocity in Figure 10. As is shown, both maximums were found in a small range of velocities, called the “process window” [42], between 2 mm/s and 6.5 mm/s for tensile modulus, and strength values in the range from 1.5 mm/s to 6.4 mm/s. In summary, tensile strength values were 191.4 ± 42.5 MPa for fibers oriented at 0°, 69.8 ± 10 MPa at 45°, and 45.7 ± 7.8 MPa at 90°. Additionally, tensile modulus values were 19.0 ± 1.4 GPa for fibers oriented at 0°, 6.2 ± 0.9 MPa at 45°, and 5.2 ± 1.1 MPa at 90°. Despite the fact that the data for all fiber orientations were fitted as a piecewise linear function, the band was fuzzy defined since many factors such as specimen preparation or fiber misalignments could affect the final result [43]. The high range of variation of the tensile strength for 0° when it is compared with the range of variation of the tensile module is to be highlighted. This could be explained considering the effect of voids as stress concentrators and crack initiators, which mainly affects strength properties, rather than elastic properties. The human factor during manufacturing is an additional and unavoidable factor which may increase these ranges. 

### 4.3. Relationship between Voids and Flow Front Velocity

**Figure 11:**
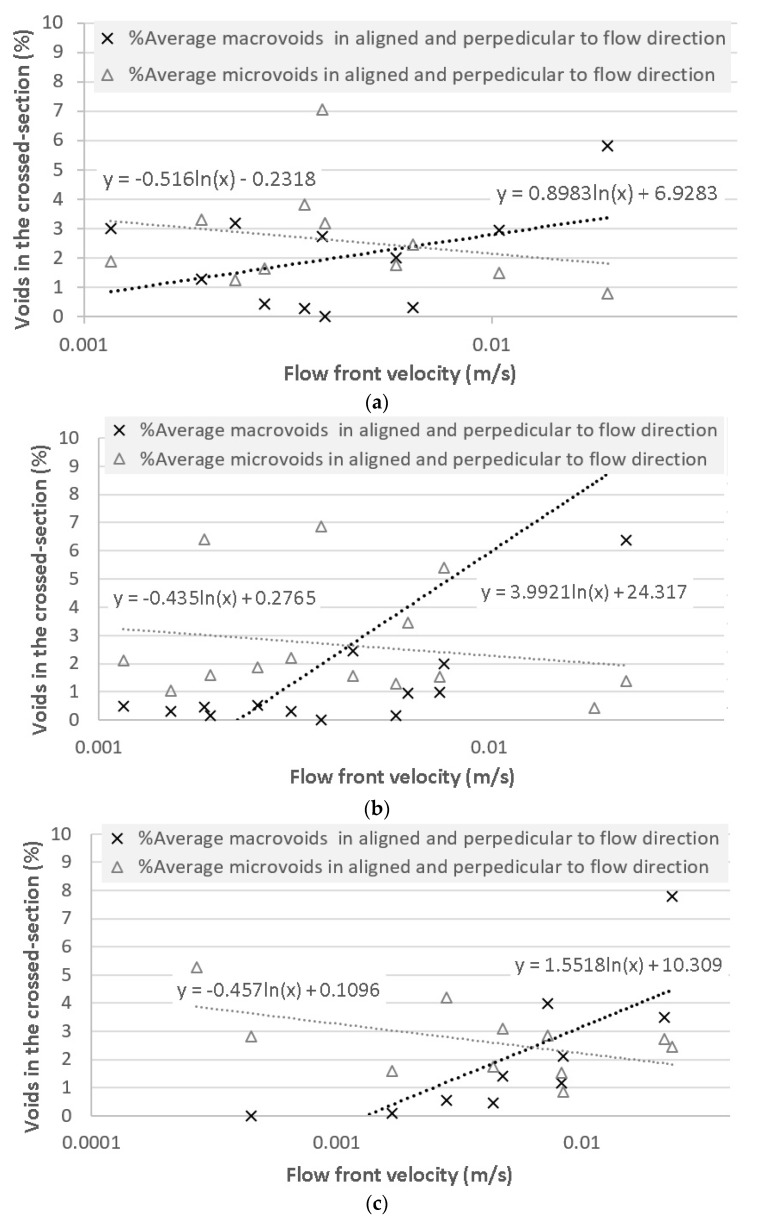
Void content as a percentage of the cross-section area for specimens cut in the parallel and the perpendicular directions, depending on the flow front velocity for all tested laminates at (**a**) 0°, (**b**) 45°, and (**c**) 90°.

Voids analysis was performed for the three different fiber orientations, in which macrovoids and microvoids were evaluated and plotted against the flow front velocity. The results are shown in Figure 11, where the three directions were analyzed separately. 

For the three tested fiber directions, the trends are quite similar. From a logarithmic fitting, it was found that the intersection of both the microvoid and macrovoid curves is within the flow velocity range of 4.38 mm/s at 45° to 6.33 mm/s at 0° and 6.24 mm/s at 90°. Consequently, no relationship was found regarding the fiber orientation angle with respect to void generation.

### 4.4. Results with the Proposed Methodology

**Figure 12:**
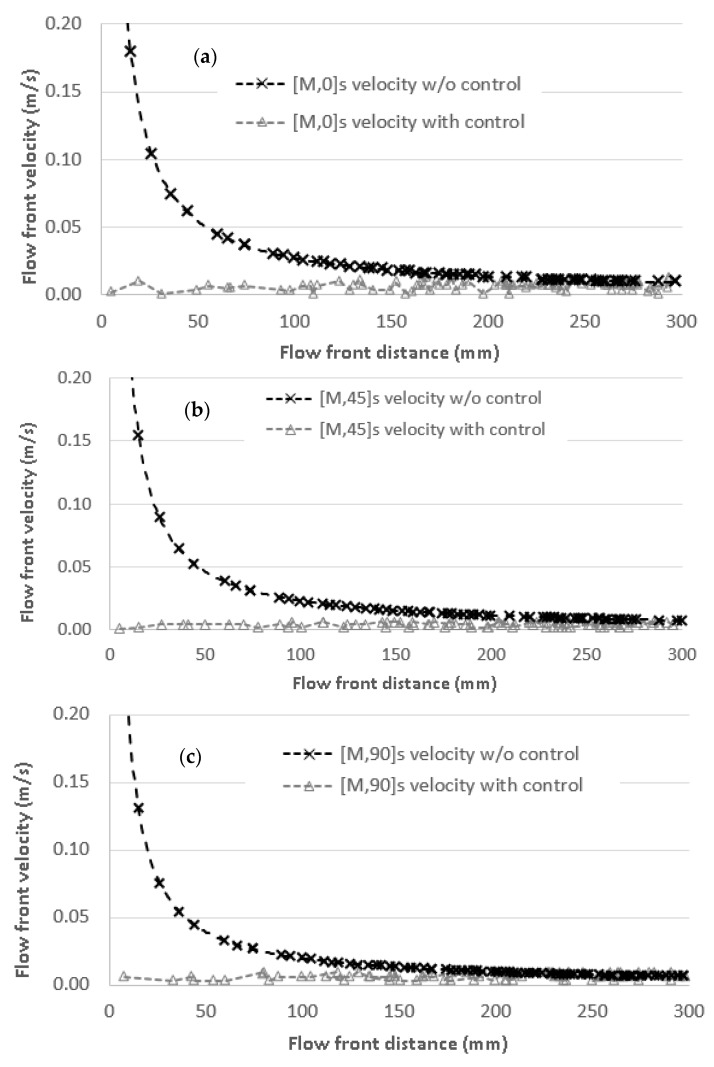
Flow front velocity as a function of flow front position for the fiber orientation at (**a**) 0°, (**b**) 45°, and (**c**) 90° with and without velocity control. In all of these cases, the non-controlled velocity was higher than the optimum velocity and the whole laminate could be impregnated at the optimum velocity.

Once the optimum values were set by the void content and tensile analysis, the impregnation process was conducted using the proposed methodology and using the optimum values found in the current study. The flow front velocities that provided the minimum void content were used for each orientation: 6.33 mm/s at 0°, 4.38 mm/s at 45°, and 6.24 mm/s at 90° as a set point in the controller to manufacture the new flow front specimens. Results are shown in Figure 12, where the three fiber orientations are compared to the non-controlled tests. Intrinsic variations in the controlled flow front velocities are generated, mainly at the beginning of impregnation as a consequence of the high pressure applied over a small length. Nevertheless, it is difficult to finely control the velocity at this first stage.

The mechanical analysis using results from tensile tests shows a tensile strength of 225.4 ± 16.0 MPa for specimens at 0°, 73.2 ± 9.1 MPa for 45°, and 47.2 ± 4.5 MPa for 90°, taking into account that these directions correspond to the cut specimens. Similar trends were observed for the elastic modulus: 20.1 ± 1.0 GPa for fibers at 0°, 6.9 ± 0.6 GPa for fibers at 45°, and 5.6 ± 0.4 GPa for fibers at 90°. These results show clear benefits of controlling the flow front velocity, increasing the tensile strength around 18.0% at 0°, 4.9% at 45°, and 3.3% at 90°, as well as the tensile modulus with increments of 18.4% for 0°, 10.8% for 45°, and 8.7% for 90°. Regarding the standard deviations, all tests showed a mean reduction of 38.2% for tensile strength and 44.9% for tensile modulus, obtaining more stable results.

## 5. Conclusions

As highlighted in this work, the air entrapment phenomenon is strongly dependent on the flow front velocity, which has a remarkable influence over tensile properties. Despite the well-known strong influence of fiber orientation on the filling time, increasing the infusion time around 50% from fibers at 0° to fibers at 90°, these effects were not noticed at the optimum velocity, which shows no clear dependence of fiber orientation. For the three tested directions, the optimum flow front velocity was in the range of 4.5 to 6.5 mm/s, although the band where maximum tensile properties were founded was slightly thicker. Following this methodology, tensile strength was enhanced up to 18% for fibers placed at 0° and 3.3% for fibers at 90°, whereas tensile modulus was increased up to 18.4% for fibers placed at 0° and 8.7% for fibers at 90°. Additionally, the standard deviations showed reductions up to 38.2% for tensile strength and 44.9% for tensile modulus.

Moreover, only one complete test using an untested material was enough to define the best impregnation velocity, which will make it possible to improve the mechanical properties in any component to be infused compared to non-controlled impregnations. This velocity can be defined in the feedback control system as a set point, and it will be guaranteed over the whole impregnation process. Through this real-time flow front velocity control, RFI processes will unavoidably reduce the influence of manual work done by workers, and mechanical properties and material quality will consequently improve. This fact implies a dual weight reduction in composite parts: improved mechanical properties means a weight reduction and the design security coefficient can be reduced due to the reduction of worker skill dependence.

Despite the improvement of void content and tensile properties using this flow front control methodology, other mechanical properties in which the matrix plays a considerable role could be also improved, such as flexural or fatigue performance. Additionally, it must be highlighted that the evolution of flow front and flow rate as a function of time could be programmed into conventional PID systems, once previous testing has been performed.

## Figures and Tables

**Table 1:** Results of filling analysis for fibers at 0°, 45°, and 90°, when valves are totally opened and maximum vacuum of −95.0 kPa is applied.

Orientation	Filling Time at Maximum Velocity (s)	Minimum Flow Front Velocity (mm/s) ^1^	$C=\frac{\Delta P\cdot K}{\mu }$ $\left(\frac{m^{2}}{s}\right)$	ΔP (kPa)	Permeability *K* (m^2^)
0°	31.0 ± 0.1	4.67	2.70 × 10^−3^	95.0	9.52 × 10^−9^
45°	40.4 ± 0.1	3.78	2.32 × 10^−3^	95.0	8.17 × 10^−9^
90°	46.2 ± 0.1	3.26	1.96 × 10^−3^	95.0	6.93 × 10^−9^

^1^ Measured at the end of laminate filling.
